# What is known from the existing literature about the treatment of Mallet Injury using 3D printed splints? A Scoping Review Protocol

**DOI:** 10.12688/hrbopenres.13865.1

**Published:** 2024-04-24

**Authors:** Una M. Cronin, Alice Shannon, Micheal ó hAodha, Aidan O'Sullivan, Niamh M. Cummins, Leonard OSullivan

**Affiliations:** 1 Rapid Innovation Unit, Confirm Centre for Smart Manufacturing, University of Limerick, Limerick, County Limerick, Ireland; 2 Health Research Institute, University of Limerick, Limerick, County Limerick, Ireland; 3 National Children's Research Centre, Crumlin, County Dublin, Ireland; 4 Faculty Librarian (Science, Engineering and Architecture), Glucksman Library, University of Limerick, Limerick, County Limerick, Ireland; 5 School of Medicine, SLÁINTE Research and Education Alliance in General Practice, Primary Healthcare and Public Health. Faculty of Education and Health Sciences,, University of Limerick, Limerick, County Limerick, Ireland; 6 Department of Paramedicine, School of Primary and Allied Health Care, Monash University, Melbourne, Australia

**Keywords:** Mallet injury, 3D printing

## Abstract

**Background:**

Mallet finger injuries are a frequent cause of hospital attendance, being the fifth most common injury in the body. They are therefore a frequent cause of hospital visits. To date, these injuries have primarily been managed using generic splints. As a generic splint provides a generic fit, patients who receive these are not provided with a custom splint experience. As the size and fit of these splints are not bespoke to the patient’s anatomy, patients may not always find the fit comfortable and may find complying with these splints difficult at times. However, an opportunity is developing within healthcare where custom splinting can be obtained for some using Three-D (3D) printing. The rationale for this review is to gain an understanding of the research that has been conducted on 3D printing of mallet injury splints.

**Objective:**

The objective of this scoping review is to map the current literature on 3D printing associated with mallet finger injury.

**Methods:**

The Joanna Briggs Institute (JBI) methodology for scoping reviews will be used throughout along with the Preferred Reporting Items for Systematic Reviews and Meta-Analyses extension for Scoping Reviews (PRISMA-ScR). Two researchers will search the databases that will include CINAHL, Embase, Cochrane, EbscoHost, Medline/Pubmed, Science Direct, Web of Science, and Google Scholar. The search will include grey literature and a hand search of sources falling outside the chosen databases. Screen titles, abstracts, and full-text articles will be reviewed by two researchers independently using Rayaan software. The data extracted from the literature will first be presented in a tabulated chart followed by a narrative synthesis.

**Registration:**

The protocol was registered on 6 ^th^ September 2023, with the Open Science Framework. Registration DOI: https://doi.org/10.17605/OSF.IO/FSJPK

## Introduction

Mallet finger is a common finger injury. It occurs when the distal interphalangeal joint (DIPj) is unable to extend, usually following trauma to the distal phalynx ^1^. In mallet injuries, the tendon is partially torn or experiences a complete rupture ^2^. An avulsion fracture can also occur. Mallet finger injuries are often caused by trauma, such as a forceful blow to the DIPj ^3^.

Uncomplicated cases of mallet injury are treated with immobilisation of the DIPj, where the joint is held in extension for 6–8 weeks using a splint ^4^. The splint must be worn continuously to keep an upward force on the injured finger until the tendon injury or fracture heals. If the splint is removed and the injured finger bends, the extensor tendon can re-rupture, meaning the splinting process needs to be repeated, which can delay healing by a number of weeks ^5^.

There are many splint variations available in healthcare facilities, with most studies reporting similar results ^6– 8^. Although no gold standard exists regarding the optimum splint to use for the treatment of mallet injury, many options are available. Some examples include generic Stack splints, aluminum foam splints, dorsal glue splints and custom thermoplastic splints ^9^.

A systematic review conducted in 2015 compared custom-made finger splints with pre-fabricated splints ^10^. They found custom splinting resulted in less skin complications as compared with custom-made splints. Another study conducted in 2022, recommended clinicians should use custom splints to best provide complete immobilisation of the DIPj which is essential to ensure recovery from the injury ^11^. Although generic and custom splints are available within some healthcare facilities, they do have documented complications. Examples of these include skin complications, the splint being bulky, not being waterproof and not being well ventilated ^12^.

However, the emerging field of 3D printing is showing promising alternatives to generic and hand moulded custom splints. 3D printing involves printing materials by reading a digital blueprint and then printing the object layer by layer. The benefits offered by 3D printing include cost effective prototyping, time efficient modelling, the manufacture of complex geometries ^13^. Most recently, 3D printing has been adapted to directly treat patients, moving away from its initial application for anatomical modelling to aid in education and planning for procedures ^14^. Advances in 3D printing technology and experience have led to increased clinical use, including in personalised healthcare solutions ^15^. There is now an opportunity to provide custom care to patients suffering from mallet injury using 3D printing. To progress this concept, it is essential to gain an understanding of the research conducted on this topic thus far.

To date, some reviews have been conducted within the domain of 3D printing splints and orthoses ^12, 16– 18^. The most relevant review to our realm of interest was conducted by Oud in 2021 ^12^. Their review primarily focused on 3D-printed orthosis for hand conditions secondary to trauma, with a concentration on their effect. They found the literature consisted of low patient numbers and had poor methodological quality. They highlighted there is an opportunity for high-quality controlled trials ^12^.

The broad scope and autonomy afforded in Scoping Reviews provide a template to identify the main concepts and themes along with the research gaps associated with 3D printing and mallet splints. As this area is a novel development, the associated literature may lack high quality and homogeneous studies. A preliminary google search uncovered relevant literature available in healthcare journals. To that end, a scoping review was chosen to allow an exploration of the breadth of published and grey literature on the topic of the treatment of mallet injury using 3D-printed splints. A holistic synthesis of the available evidence will be conducted, while locating gaps in the research and highlighting areas for further research focus.

## Aim and review question

This scoping review aims to deliver a thorough comprehension of all the research available on the topic of 3D-printing of mallet splints. The JBI methodology for scoping reviews will be utilised to conduct this scoping review ^19^.

The Population Concept Context (PCC) framework guided the development of the main research questions as follows:

### Participants

Eligible studies will include participants of any age with mallet injury due to trauma.

### Concept

The phenomenon of interest is 3D printing of mallet splints.

### Context

As 3D printing of mallet splints is a novel and emerging field, we felt it important to include both clinical and research settings. The preliminary search of the literature identified some studies detailing the design and print of mallet splints not designed for or placed on specific patients with mallet injuries. These studies describe the process and science emerging around 3D printing of mallet splints in the research setting, thus will be included as they will add a further layer of experience to the clinical studies.

The sub-questions underpinning this overarching question include:

1. What techniques and processes (examples being measuring, printing, securing) are most frequently used for 3D printing mallet splints on patients and in research settings?
2. What is the potential for 3D printing of mallet splints in the clinical field/ point of care?

## Eligibility criteria

Inclusion criteria

- Studies that report on 3D printing of mallet splints or 3D printing for mallet injury.
- Literature published since 1980. The earliest applications of 3D printing were in the 1980’s therefore the search will range from 1980 to end of September 2023 ^20^.
- Only full-text publications will be included.
- Articles published in English only.
- Grey literature including studies and reports that mention 3D printing of mallet splints.

Exclusion criteria

- There are no specific exclusion criteria.

### Types of sources

All study methodologies will be considered in this review. Both qualitative and quantitative data will also be included in the review. Text and opinion papers will also be considered for inclusion in this scoping review.

## Methods

A preliminary search of Embase, CINAHL, Cochrane, PUBMED (Medline), Google Scholar and JBI Evidence Synthesis was conducted on September 6 ^th^ 2023. No past or ongoing scoping reviews on this subject were discovered.

The review was conducted using the JBI methodology guidance for scoping reviews. The protocol was registered on the 6 ^th^ of September 2023 with the Open Science Framework.

### Search strategy

The search strategy will be created in association with a specialist librarian (MOH). The review will include both published and unpublished literature. The search strategy will be iterative throughout with the first step involving a literature search of the subsequent databases: CINAHL, EMBASE, Science Direct, Cochrane, Web of Science, MEDLINE (Pubmed), and Google Scholar. The search strategy will involve the combining of medical subject headings (MESH) and Boolean Operators. Identified keywords and English language filters will be applied. Each indexed database will include an adapted search strategy. Following this, the reference lists of each included study will be examined. The authors of eligible studies will be contacted as needed for extra material or to clarify any unclear areas in the literature. The published review will include the entire search strategy and results. The search strategy is presented in Table 1.

**Table 1.** Proposed search strategy.

Database	Exact search
Ebscohost	mallet AND ("additive manufacturing" or "3d printing" or "3-d printing" or "three-dimensional printing")
EMBASE	mallet AND ("additive manufacturing" or "3d printing" or "3-d printing" or "three-dimensional printing")
Scopus	mallet (title, abstract, keywords) AND ("additive manufacturing" or "3d printing" or "3-d printing" or "three-dimensional printing") (title, abstract, keywords)
Pubmed	mallet AND ("additive manufacturing" or "3d printing" or "3-d printing" or "three-dimensional printing")
Web of Science	mallet AND ("additive manufacturing" or "3d printing" or "3-d printing" or "three-dimensional printing")
Science direct	mallet (title, abstract, keywords) AND ("additive manufacturing" or "3d printing" or "3-d printing" or "three-dimensional printing") (title, abstract, keywords)

### Study/source of evidence selection

After completing the search, all relevant references will be inserted post-collating into Endnote X8 (Clarivate Analytics, PA, USA). The screening process will be carried out using Rayaan, a web-based literature screening program. Duplicates will then be deleted. Two reviewers (UC and AS) will conduct a pilot test (n=5) by screening the title and abstracts of the search using the set eligibility criteria. Following this, the full manuscript of each chosen literature will be reviewed using the predetermined eligibility criteria. The sources of evidence excluded at full text will be recorded and detailed in this review. A ‘snowball’ search methodology will be adopted by reviewing the reference lists of the chosen studies to aid in identifying any literature that was not captured in the initial search. Any conflicts in relation to inclusion of specific literature will be discussed, and if not resolved, a third reviewer (NC) will be consulted. All outcomes of the literature search and the process of inclusion and exclusion will be presented clearly in the final paper.

### Data extraction

A data extraction tool will be created by the researchers to aid the information extraction process. The extracted data shall provide detail on the participants, concept and context of each paper. The study methodologies utilised, along with all data relevant to the review question, what is known from the existing literature regarding the treatment of mallet injury using 3D-printed splints will be presented.

Table 2 displays the initial draft data extraction tool created by two researchers. As is typical for scoping reviews, the data extraction tool will be modified as needed throughout the iterative process of information review and extraction. Any modifications made to the data extraction tool will be presented clearly in the final review. Again, any conflicts in decisions regarding inclusion and exclusion of data that arise will be discussed and resolved if needed by a third reviewer. If additional information is needed, the relevant authors of the paper will be communicated with to provide clarity or supplementary data. The purpose of the scoping review is to describe current practices relating to the 3D-printing of mallet splints thus a critical appraisal of the methodological quality of each paper is not essential.

**Table 2.** Adapted data charting form from Joanna Briggs Institute (JBI) methodology for scoping reviews ^16^.

Citation details
Inclusion/Exclusion criteria
Context
Study design (RCT etc)
Study populations/sample size/injured, non-injured
Methodology
Adverse events
Endpoints
Limitations
Specifics of splints
Sustainability
How secured
How measured
Printer/material used
Mention of compliance
Process used
Skin integrity/ventilation

### Data analysis and presentation

All data will first be introduced in a descriptive manner, using tables and charts to aid the explanation of the results. A narrative summary will also be included. This approach ensures a logical and descriptive summary that conforms with the objective of scoping reviews. The PRISMA-ScR will be used to guide the report ^21^.

**Registration**: The protocol was registered on 6 ^th^ September 2023, with the Open Science Framework. Registration DOI: https://doi.org/10.17605/OSF.IO/FSJPK

## Data Availability

No data are associated with this article.
