# Supplementary material for: What is known from the existing literature about the treatment of Mallet Injury using 3D printed splints? A Scoping Review Protocol
Source: HRB Open Res. 2024 Aug 16;7:21. Originally published 2024 Apr 24. [Version 2] doi: 10.12688/hrbopenres.13865.2 (PMC11415759; doi:10.12688/hrbopenres.13865.2)
Supplement: Peer review history

# Reviewer report: Reviewer response for version 2

**DOI:** 10.21956/hrbopenres.15295.r42446

**Published:** 2024-09-20

**Authors:** Yutaro Kuwahara

**Affiliations:** 1 Toyohashi Municipal Hospital, Aichi, Japan

Dear Author

 I am honored to have the opportunity to review this protocol study. As with your opinions, I basically prefer to surgical procedures such as pinning and pull-out methods due to the lack of suitable splints and the lack of access to 3D printers at my place. I consider that the methodology and clinical questions of this study are adequately described, so I consider it “Accepted”. I would like to make a few minor modifications and suggestions below.

**Introduction**

 “They found custom splinting resulted in less skin complications as compared with custom-made splints. “

 Do you mean custom-made splints resulted in less skin complications compared with ready-made splints?

**Data extraction**

 Although it may be included in the endpoints, it is more interesting to analyze data on postoperative range of motion and recurrence rate.

 I would like to thank you again for giving me this valuable chance.

 Sincerely,

Is the study design appropriate for the research question?

Yes

Is the rationale for, and objectives of, the study clearly described?

Yes

Are sufficient details of the methods provided to allow replication by others?

Yes

Are the datasets clearly presented in a useable and accessible format?

Yes

Reviewer Expertise:

Orthopaedic surgery, Hand surgery, Trauma surgery

I confirm that I have read this submission and believe that I have an appropriate level of expertise to confirm that it is of an acceptable scientific standard.

# Reviewer report: Reviewer response for version 2

**DOI:** 10.21956/hrbopenres.15295.r41988

**Published:** 2024-08-30

**Authors:** Ryan Trickett

**Affiliations:** 1 University Hospital of Wales, Cardiff, UK

The authors have acknowledged my queries and responded. I still consider that the protocol as described is narrow in scope, but will provide decisive answers concerning this somewhat niche field.  

 I also acknowledge that there are as yet no results or conclusions to comment on in this report.

Is the study design appropriate for the research question?

Yes

Is the rationale for, and objectives of, the study clearly described?

Yes

Are sufficient details of the methods provided to allow replication by others?

Partly

Are the datasets clearly presented in a useable and accessible format?

Partly

Reviewer Expertise:

Hand trauma and surgery

I confirm that I have read this submission and believe that I have an appropriate level of expertise to confirm that it is of an acceptable scientific standard.

# Reviewer report: Reviewer response for version 1

**DOI:** 10.21956/hrbopenres.15195.r41020

**Published:** 2024-07-11

**Authors:** Ryan Trickett

**Affiliations:** 1 University Hospital of Wales, Cardiff, UK

In the introduction the authors discuss mallet finger and avulsion fractures. It is important to be clear that soft tissue mallet fingers and TRUE avulsion fractures are likely very similar injuries in terms of mechanism and outcome – true hyperflexion injuries and usually relatively low energy trauma.

 So-called bony mallet fractures, particularly those involving a larger proportion of the articular surface are much more likely to be an alternative mechanism – axial loading associated with EXTENSION of the distal joint – with a greater force of injury. These injuries conversely are often best treated with slight flexion, particularly where there is articular incongruence. All mallet injuries are often clumped together in the literature but should be clearly defined in the introduction and the inclusion criteria should be clear in this regard too.

 Participants – soft tissue and/or bony and/or hyperextension injuries (see above)?

 Why has September 2023 been chosen as an end date to the searches?

 Can the authors be more specific around their searching of the “grey literature"? It is also odd that only “full-text” articles are being included, but grey literature is being included. The 2 statements seem at odds with one another.

 Has chronicity of mallet finger been considered in the inclusion criteria?

 Why have non-English articles been excluded? This potentially adds a selection bias.

 I worry that the search will yield no usable outcomes data and simply demonstrate what we almost certainly know already – that there is an almost complete lack of literature around 3D printing of mallet splints. A quick search using the search strategy – which seems to be somewhat restrictive – shows only 2 relevant articles.

 Why was mallet finger chosen specifically for this review?

 Perhaps a more useful review could be “3D printing splints for the treatment of closed digital tendon injuries” which could include mallets and Boutonniere’s; or an even wider scope to include open tendon injuries?

Is the study design appropriate for the research question?

Yes

Is the rationale for, and objectives of, the study clearly described?

Yes

Are sufficient details of the methods provided to allow replication by others?

Partly

Are the datasets clearly presented in a useable and accessible format?

Partly

Reviewer Expertise:

Hand trauma and surgery

I confirm that I have read this submission and believe that I have an appropriate level of expertise to confirm that it is of an acceptable scientific standard, however I have significant reservations, as outlined above.

# Response

**Published:** 2024-07-17

**Authors:** Leonard OSullivan

**Affiliations:** Health Research Institute, University of Limerick, Castletory, Limerick, Ireland

**Comment 1:** In the introduction the authors discuss mallet finger and avulsion fractures. It is important to be clear that soft tissue mallet fingers and TRUE avulsion fractures are likely very similar injuries in terms of mechanism and outcome – true hyperflexion injuries and usually relatively low energy trauma.

 So-called bony mallet fractures, particularly those involving a larger proportion of the articular surface are much more likely to be an alternative mechanism – axial loading associated with EXTENSION of the distal joint – with a greater force of injury. These injuries conversely are often best treated with slight flexion, particularly where there is articular incongruence. All mallet injuries are often clumped together in the literature but should be clearly defined in the introduction and the inclusion criteria should be clear in this regard too.

**Response 1:** Thank you for your comments. Your direction is much appreciated.

 The following updates have been made to the text:

 Mallet finger is a common finger injury. It occurs when the distal interphalangeal joint (DIPj) is unable to extend, usually following trauma to the distal phalynx. In soft tissue mallet injuries, the tendon is partially torn or experiences a complete rupture, usually following a low energy trauma. Bony mallet injuries, whereby a large proportion of the articular surface is involved, is associated with extension of the distal joint, following a greater force of injury.

**Comment 2:** Participants – soft tissue and/or bony and/or hyperextension injuries (see above)?

**Response 2:** Based on your suggestion, this text has been updated to: Participants: Eligible studies will include participants of any age with soft tissue and/or bony and/or hyperextension mallet injury due to trauma.

**Comment 3**: Why has September 2023 been chosen as an end date to the searches?

**Response 3:** The plan for the search strategy was based on academic needs.

****

**Comment 4:** Can the authors be more specific around their searching of the “grey literature"? It is also odd that only “full-text” articles are being included, but grey literature is being included. The 2 statements seem at odds with one another.

**Response 4:** Gray literature will be removed from the inclusion criteria.

**Comment 5:** Has chronicity of mallet finger been considered in the inclusion criteria?         

**Response 5:** This is a helpful suggestion. Both acute and chronic mallet injuries will be included in the criteria.

**Comment 6:** Why have non-English articles been excluded? This potentially adds a selection bias. 

**Response 6:** Resource constraints within the research team preclude us from translating any literature outside of English.

**Comment 7:** I worry that the search will yield no usable outcomes data and simply demonstrate what we almost certainly know already – that there is an almost complete lack of literature around 3D printing of mallet splints. A quick search using the search strategy – which seems to be somewhat restrictive – shows only 2 relevant articles.                                                                                                                         

**Response 7:** This is a reasonable suggestion, thank you. As this work is contributing toward a PhD for the author, it was deemed important to take the opportunity to delve into any literature related to mallet injury and 3D printing, to give a thorough analysis on which to base the future studies on.

**Comment 8:** Why was mallet finger chosen specifically for this review?

**Response 8:** This study contributed towards a PhD for the author, and their area of focus in 3D printing and mallet injuries.

**Comment 9:** Perhaps a more useful review could be “3D printing splints for the treatment of closed digital tendon injuries” which could include mallets and Boutonniere’s; or an even wider scope to include open tendon injuries?                                                                                                                         

**Response 9:** This is a reasonable suggestion. The language in participants has been updated to better explain the scope of the inclusion: Participants: Eligible studies will include participants of any age with soft tissue and/or bony and/or hyperextension mallet injury due to trauma.
